# Endoplasmic Reticulum Stress: A Novel Target for the Prevention and Treatment of Hypertension and Its Related Diseases

**DOI:** 10.1111/jcmm.70977

**Published:** 2025-12-08

**Authors:** Xin Ma, Fei Si, Jie Ma, Chuyan Feng, Yingdong Wang, Luosha Wang, Jing Yu

**Affiliations:** ^1^ The Second Hospital & Clinical Medical School Lanzhou University Lanzhou China

**Keywords:** endoplasmic reticulum stress, ERS‐related signalling pathways, hypertension, pathogenic mechanisms, targeted therapy

## Abstract

Endoplasmic reticulum stress (ERS) emerges as a critical pathophysiological nexus in hypertension and related cardiovascular diseases. Chronic ERS activation via the IRE1α‐XBP1, ATF6, and PERK pathways drives vascular endothelial dysfunction (reduced NO bioavailability, increased ET‐1), renin‐angiotensin system (RAS) hyperactivation, sympathetic overactivation, and vascular smooth muscle cell (VSMC) maladaptive proliferation/apoptosis, collectively promoting hypertension progression and end‐organ damage. Pharmacological targeting of ERS demonstrates therapeutic promise: chemical chaperones 4‐phenylbutyric acid (4‐PBA) and tauroursodeoxycholic acid (TUDCA) stabilise proteostasis, reduce oxidative stress, and inhibit apoptosis; antioxidants N‐acetylcysteine (NAC) and melatonin attenuate ERS‐oxidative stress crosstalk. Notably, conventional antihypertensives—ACE inhibitors and angiotensin receptor blockers (ARBs)—exert ancillary benefits by suppressing ERS beyond their primary RAS blockade. Preclinical evidence supports the efficacy of these strategies in reversing hypertensive pathophysiology. Future research must prioritise isoform‐selective ERS modulator development, validation in human trials, biomarker discovery, and elucidating ERS roles in therapy‐induced hypertension. Targeting ERS represents a transformative mechanotherapeutic paradigm for precision hypertension management.

**Abbreviations**
4‐PBA: 4‐phenylbutyric acid
ACEIs: angiotensin‐converting enzyme inhibitors
Ang‐II: angiotensin II
ARBs: angiotensin receptor blockers
AT1R: angiotensin type 1 receptor
ATF4: activating transcription factor 4
ATF6: activating transcription factor 6
BNP: brain natriuretic peptide
CHOP: C/EBP homologous protein
EndMT: endothelial–mesenchymal transition
ER: endoplasmic reticulum
ERS: endoplasmic reticulum stress
GRP78: glucose‐regulated protein 78
IRE1α: inositol‐requiring enzyme 1α
LVEF: left ventricular ejection fraction
MT: melatonin
NAC: N‐Acetylcysteine
NF‐κB p65: nuclear factor‐κB p65
PERK: protein kinase RNA‐like ER kinase
RAS: renin‐angiotensin system
ROS: reactive oxygen species
RVLM: rostral ventrolateral medulla
SHR: spontaneously hypertensive rat
SNS: sympathetic nervous system
TMAO: Trimethylamine N‐oxide
TUDCA: tauroursodeoxycholic acid
UPR: unfolded protein response
VEGFA: vascular endothelial growth factor A
VSMCs: vascular smooth muscle cells
XBP1: X‐box binding protein 1

## 1 Introduction

Hypertension represents a predominant determinant of cardiovascular mortality and maintains its status as a globally pervasive cardiovascular pathology [1, 2]. In China, hypertension persists as the most prevalent chronic non‐communicable disease and constitutes the principal etiological factor underlying cardiovascular‐related mortality across urban and rural populations [3]. The clinical burden of hypertension stems from its pervasive impact on end‐organ function. This manifests as impaired endothelial‐dependent vasodilation and increased peripheral vascular resistance in the arterial system [4]; dysregulated pressure‐natriuresis and glomerular hypertension in the kidneys, often progressing to fibrosis [5]; compensatory left ventricular hypertrophy that may evolve into diastolic dysfunction and heart failure in the myocardium [6]; and altered cerebral autoregulation and vascular remodelling in the cerebrovasculature, significantly elevating the risk of stroke and cognitive impairment [7]. These deleterious physiological changes are ultimately driven by underlying molecular and cellular disturbances, highlighting the critical need to elucidate the fundamental mechanisms, such as endoplasmic reticulum stress (ERS), that orchestrate these pathological processes.

The endoplasmic reticulum (ER), a principal organelle in eukaryotic systems, orchestrates protein biogenesis and fidelity control, intracellular vesicular trafficking and systemic proteostasis [8], while concurrently functioning as a central regulator of intracellular calcium flux dynamics [9]. Under conditions of pathophysiological perturbation or metabolic dysregulation, the accrual of misfolded polypeptides within the ER lumen induces proteostatic imbalance, thereby instigating ERS [10]. Evolutionary adaptation has endowed the ER with a tripartite transmembrane signal transduction apparatus comprising inositol‐requiring enzyme 1α (IRE1α), activating transcription factor 6 (ATF6), and protein kinase RNA‐like ER kinase (PERK) to detect ERS [11]. These molecular sentinels initiate the unfolded protein response (UPR) [12], an evolutionarily conserved homeostatic program mediating restoration of ER proteostatic equilibrium. The pathophysiological significance of ER stress is increasingly recognised in diverse cardiovascular diseases. Furthermore, its observed activation in hypertensive target organ damage suggests a pivotal mechanistic driver in disease progression [13]. This analytical review systematically delineates the multiscale mechanisms through which ERS modulates hypertensive pathogenesis, identifies molecular targets within ERS regulatory networks, and proposes innovative therapeutic frameworks to advance preventive and interventional strategies for hypertension management.

## 2 Overview of ERS Related Pathways

### 2.1 The IRE1α Signalling Pathway

IRE1α (inositol‐requiring enzyme 1α), an evolutionarily conserved transducer of ERS, functions as a type I transmembrane protein anchored within the ER membrane. Its molecular architecture exhibits a bipartite structural organization [14]: an N‐terminal luminal domain mediating unfolded protein recognition, and a C‐terminal cytosolic domain harbouring dual enzymatic functionalities—serine/threonine kinase activity and site‐specific ribonuclease capacity, both essential for orchestrating downstream signal transduction cascades [15]. Under homeostatic conditions, IRE1α maintains a quiescent monomeric conformation through complexation with the ER‐resident chaperone glucose‐regulated protein 78 (GRP78/BiP). During ERS induction, GRP78 dissociation facilitates IRE1α homodimerization [16], followed by autocatalytic trans‐autophosphorylation that induces conformational activation of its ribonuclease module [17]. The activated cytoplasmic domain then executes unconventional splicing of X‐box binding protein 1 (XBP1) mRNA, generating the potent transcription factor spliced XBP1 (XBP1s), which drives the expression of genes essential for restoring ER proteostasis [18].

Notably, the IRE1α signalling axis elicits dichotomous pathophysiological outcomes, pivoting between adaptive and pro‐apoptotic responses contingent upon the cellular context. This functional divergence is mediated through its distinct downstream effectors. The IRE1α‐XBP1s branch primarily orchestrates adaptive cellular programs, which under specific conditions can drive pathophysiological processes such as microglial activation, epithelial‐mesenchymal transition modulation, and the upregulation of vascular endothelial growth factor A (VEGFA) to potentiate angiogenesis [19, 20, 21, 22]. Conversely, suppression of this adaptive arm, for instance through downregulation of stress‐responsive nucleoprotein‐1, effectively inhibits VEGFA expression and attenuates angiogenic signalling [22], illustrating the context‐dependent nature of this pathway. Under persistent and irremediable stress, IRE1α signalling pivots to pro‐apoptosis through its kinase domain, recruiting Apoptosis signal‐regulating kinase 1 (ASK1) to activate the c‐jun N‐terminal kinase (JNK) and p38MAPK cascades [23]. These pathways converge on the transcriptional upregulation of the C/EBP homologous protein (CHOP), a key mediator of ERS‐induced apoptosis. The deleterious impact of this IRE1α‐JNK‐CHOP axis is evidenced by its capacity to induce caspase‐dependent cell death and impair regenerative processes, as demonstrated in models of porcine embryonic development [24] and hepatic lipotoxicity [25]. Pathologically, the relevance of this pathway to hypertension is highlighted by findings that angiotensin II stimulates the IRE1α‐ASK1‐JNK‐p38MAPK signalling nexus, contributing to vascular dysfunction [23].

### 2.2 The ATF6 Signalling Pathway

ATF6 (activating transcription factor 6), a type II transmembrane protein residing within the ER, constitutes a member of the basic leucine zipper (bZIP) transcription factor superfamily. Under conditions of ERS, ATF6 undergoes retrotranslocation to the Golgi apparatus, where it undergoes site‐specific proteolytic maturation to generate its transcriptionally active nuclear form, thereby initiating downstream adaptive signalling cascades [26]. This transcription factor orchestrates critical cellular processes, including the regulation of autophagic flux and apoptotic signalling [27]. The downstream effects of ATF6 are complex and context‐dependent. Its signalling axis can promote cellular adaptation and enhance differentiation potential under certain conditions [28]; however, persistent activation of the ATF6‐CHOP branch invariably shifts the cellular outcome towards cell cycle arrest and potentiation of ERS‐mediated apoptosis [29]. The pathophysiological significance of ATF6 signalling is evidenced by its involvement in metabolic and inflammatory stress responses across various tissues [30, 31, 32, 33]. Of direct relevance to this review, targeted suppression of the ATF6 pathway has been demonstrated to ameliorate hypertension‐associated cardiac fibrotic remodelling and hemodynamic dysregulation, highlighting its central role in hypertensive end‐organ damage [34].

### 2.3 The PERK Signalling Pathway

PERK (protein kinase RNA‐like endoplasmic reticulum kinase), a type I transmembrane protein anchored within the ER, is regulated through dynamic interactions with the molecular chaperone GRP78/BiP. Under ERS, PERK undergoes autoactivation and mediates its primary function through the catalytic phosphorylation of eukaryotic initiation factor 2α (eIF2α) [35]. This event attenuates global protein synthesis while selectively promoting the translation of activating transcription factor 4 (ATF4), which amplifies a downstream gene network critical for stress adaptation [36]. The PERK‐eIF2α‐ATF4 axis constitutes a major branch of the UPR, and its activation has been documented in diverse pathophysiological contexts, including the regulation of VEGFA expression and vascular remodelling [22, 37], as well as responses to viral infection and metabolic stressors [38, 39]. Sustained activation of this pathway is a potent driver of pathological outcomes, notably the loss of microvascular integrity and the induction of Endothelial‐to‐Mesenchymal Transition (EndMT) [40]. Critically, this pathway is implicated in hypertensive vascular dysfunction, where metabolites such as trimethylamine N‐oxide (TMAO) activate the PERK‐eIF2α‐ATF4‐CHOP axis, exacerbating endothelial dysfunction and compromising vascular integrity—effects that are mitigated by PERK downregulation [39].

**FIGURE 1:**
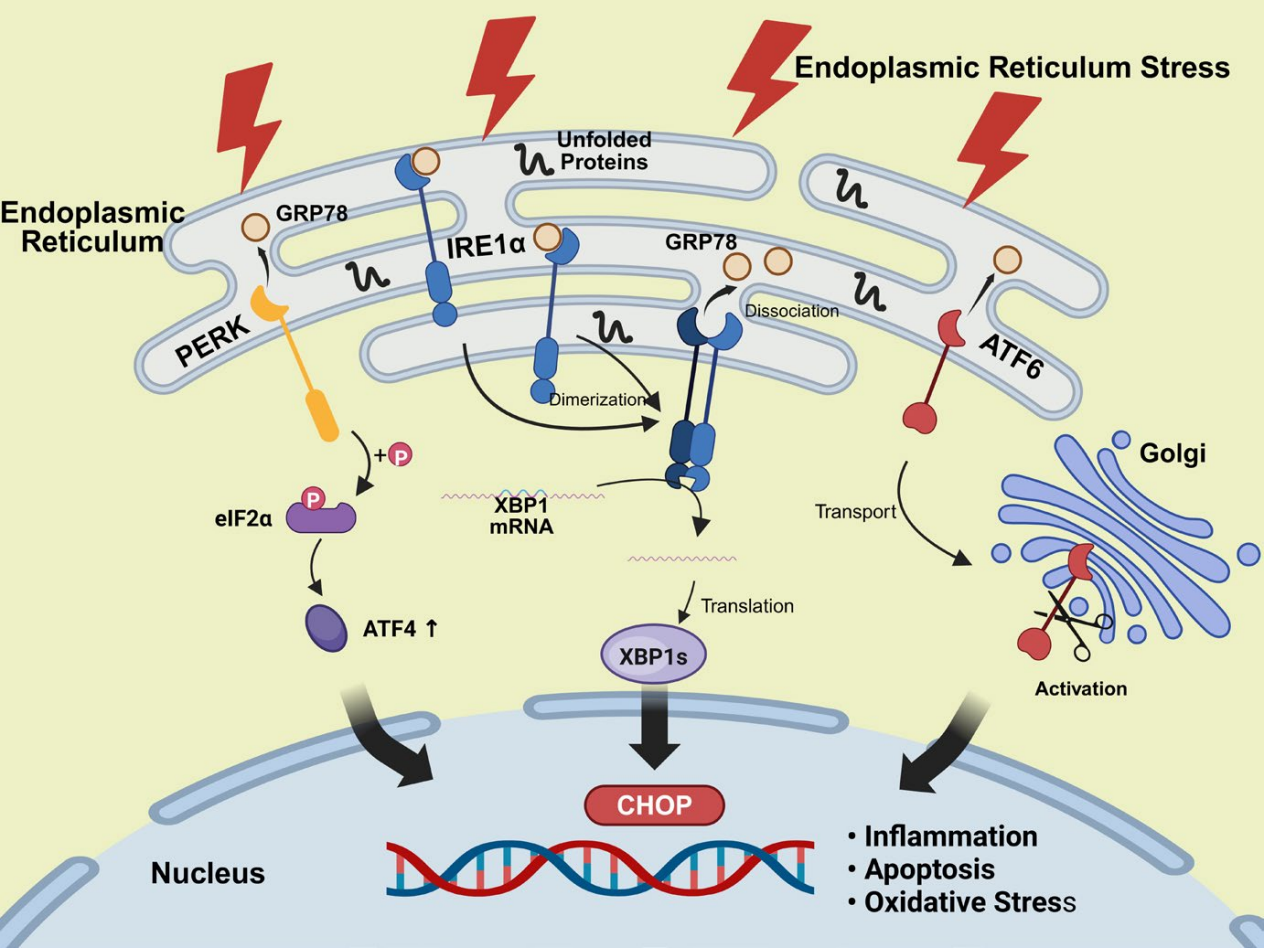
ERS related signalling pathways. Triggered by the accumulation of unfolded proteins, endoplasmic reticulum stress (ERS) is detected by three transmembrane sensors following the dissociation of the chaperone GRP78. This event activates the core ERS pathways: IRE1α dimerizes to splice XBP1 mRNA, producing the transcription factor XBP1s; ATF6 traffics to the Golgi for proteolytic activation; and PERK phosphorylates eIF2α to promote ATF4 translation. Under irremediable stress, the signalling shifts from adaptation to a pro‐apoptotic outcome, thereby driving inflammation, oxidative stress, and programmed cell death. ATF4, Activating Transcription Factor 4; ATF6, Activating Transcription Factor 6; CHOP, C/EBP Homologous Protein; eIF2α, Eukaryotic Initiation Factor 2α; GRP78, Glucose Regulated Protein 78; IRE1α, Inositol‐Requiring Enzyme 1α; PERK, Protein Kinase R (PKR)‐like Endoplasmic Reticulum Kinase; XBP1, X‐box Binding Protein 1.

Transient ERS activation confers augmented cellular adaptive resilience against pathophysiological perturbations. However, sustained or intense ERS exceeding the proteostatic resilience threshold instigates programmed cellular demise through multimodal execution pathways [41]. This dichotomous transition from cytoprotective homeostasis to cytotoxic decompensation underscores the imperative requirement for spatiotemporally constrained regulation of ERS signalling cascades. Precision therapeutic targeting of these evolutionarily conserved stress‐response mechanisms may constitute a transformative intervention paradigm for attenuating hypertensive pathophysiology and its multiorgan comorbidities (Figure 1).

## 3 The Mechanism of ERS in Hypertension and Related Diseases

### 3.1 ERS‐Induced Vascular Endothelial Dysfunction

Vascular endothelial cells orchestrate the dynamic regulation of vascular tone through paracrine secretion of vasodilatory mediators, including nitric oxide (NO) and prostacyclin (PGI_2_), coupled with transcriptional modulation of vasoconstrictive factors such as endothelin‐1 (ET‐1). This bidirectional regulation sustains endothelial‐dependent vasorelaxation and critically governs peripheral vascular resistance and hemodynamic homeostasis [42]. Endothelial oxidative stress disrupts the homeostatic equilibrium of the eNOS‐NO/ET‐1 signalling axis, precipitating vasomotor dysregulation and endothelial cytopathy—pathophysiological hallmarks central to hypertensive disease progression [43, 44].

A growing body of evidence positions ERS as a pivotal upstream mechanism driving this endothelial impairment. Endothelial injury arises not merely from redox imbalance but also through ERS‐mediated activation of the UPR, culminating in caspase‐dependent apoptotic cascades [45, 46]. Mechanistically, ERS exacerbates endothelial dysfunction through multiple convergent pathways: diminished NO bioavailability, amplified oxidative stress burden, and disrupted vascular redox signalling [47]. Pathologically, ERS activation upregulates ET‐1 transcriptional activity in endothelial cells, compromising vascular barrier integrity [48]. This link is substantiated by findings that palmitic acid induces ET‐1 overexpression in vascular endothelia via ERS activation [49]. Furthermore, ERS serves as a common pathological endpoint for diverse insults; it is implicated in the vascular toxicity of environmental plasticizers, which activate the IRE1α‐CHOP axis in endothelia [50], and in the pathophysiology of iatrogenic hypertension associated with certain pharmacotherapies [51]. Cumulatively, these findings establish ERS as a central regulator of endothelial pathophysiology and a compelling therapeutic target in hypertension.

### 3.2 Interaction Between ERS and the Renin‐Angiotensin System (RAS)

The renin‐angiotensin system (RAS), a phylogenetically conserved hormonal cascade, plays a cardinal role in the pathomechanisms of cardiovascular pathologies. Emerging evidence establishes that RAS dysregulation, particularly the hyperactivity of its effector angiotensin II (Ang II), directly instigates ERS across various cell types, thereby mechanistically contributing to hypertensive disorders [52]. This activation occurs through several key pathways: Ang II potentiates macrophage ERS through IRE1α phosphorylation‐dependent pathways, thereby accelerating inflammatory responses and atherosclerotic plaque progression [53]. Conversely, the RAS also encompasses protective arms that mitigate ERS. For instance, Angiotensin‐(1–9) [Ang‐(1–9)], an endogenous RAS‐derived peptide, confers cytoprotection against ERS‐mediated apoptotic endothelial clearance, and its exogenous administration attenuates endothelial apoptosis in hypertensive models [54]. Crucially, this interaction exhibits bidirectionality, forming a deleterious positive feedback loop. Beyond being a consequence of RAS activation, ERS itself can reciprocally amplify the systemic and local RAS. In rodent heart failure paradigms, ERS drives cerebral RAS activation via MAPK‐dependent signalling cascades, exacerbating neurogenic components of hypertension and maladaptive cardiac remodelling [55]. Furthermore, ERS contributes to Ang II‐induced endothelial dysfunction by disrupting redox homeostasis, thereby intensifying vascular damage [47]. This vicious cycle of mutual reinforcement between RAS and ERS establishes a core mechanism that propagates and amplifies hypertensive pathophysiology.

### 3.3 ERS‐Induced Sympathetic Overactivation

Chronic sympathetic hyperactivation constitutes a principal pathogenic mechanism underlying hypertension progression and compromises therapeutic responsiveness in refractory blood pressure management [56]. ERS has emerged as a critical mechanistic link in this process, operating through both central and peripheral pathways to amplify sympathetic drive. ERS exerts regulatory influences on sympathetic nervous system (SNS) circuitry through neuroimmune crosstalk [57] and direct modulation of neuronal function, thereby amplifying neuroinflammatory cascades and neurodegenerative processes implicated in neurogenic hypertension etiology [58].

The central integration of this pathway occurs notably in the rostral ventrolateral medulla (RVLM), where ERS promotes microglial activation and neuroinflammation, leading to enhanced sympathetic outflow [59]. This enhanced sympathetic outflow elevates peripheral vascular resistance primarily via α1‐adrenergic receptor‐mediated vasoconstriction and impairs endothelium‐dependent vasodilation, a process linked to reduced nitric oxide bioavailability stemming from increased reactive oxygen species [60]. Complementing this central mechanism, ERS also operates at the peripheral level, where psychogenic stressors induce ERS within SNS ganglia, precipitating calcium dysregulation and aberrant neurotransmission that further amplifies sympathetic tone [61].

The functional significance of the ERS‐SNS axis is substantiated by multiple intervention models demonstrating coordinated modulation of both systems: bromocriptine‐QR reduces central sympathetic drive through transcriptional suppression of ERS mediators [62]; carotid baroreceptor stimulation achieves blood pressure control via concurrent aortic ERS suppression and SNS inhibition [63]; stellate ganglion ablation confers vascular protection through dual regulation of ERS signalling and sympathetic efferent suppression [64]; and sleeve gastrectomy produces antihypertensive effects partially via hypothalamic ERS attenuation with concomitant neuroinflammatory pathway inhibition [65]. Collectively, these findings establish ERS as a pivotal regulator within the neuro‐cardiovascular interface, orchestrating sympathetic hyperactivity and its hemodynamic consequences in hypertension.

### 3.4 ERS Regulation of Vascular Smooth Muscle Cell Fate

Hypertensive pathogenesis involves maladaptive hyperproliferation and phenotypic remodelling of vascular smooth muscle cells (VSMCs), processes mechanistically connected to ERS activation. ERS influences VSMC fate through diverse molecular initiators and signalling pathways, ultimately contributing to vascular dysfunction. The canonical ERS chaperone GRP78 is upregulated in VSMCs within the arterial fibrous cap in atherosclerotic models, indicating sustained ERS activation in vascular pathology [66]. Mechanistically, ERS promotes VSMC apoptosis through pathways such as cholesterol‐mediated proteotoxic stress, which generates pathological ROS production and drives caspase‐dependent apoptosis [67]. Conversely, ERS can also stimulate proliferative and inflammatory pathways in VSMCs. This is evidenced by circular RNA‐mediated m6A‐dependent ERS signalling that modulates pyroptotic pathways [68], and by Lipocalin‐2, which functions as a novel ERS potentiator by augmenting intracellular iron to accelerate proliferative cascades [69]. Furthermore, both pharmacological and biomechanical stimuli can engage ERS in VSMCs. Experimentally, tunicamycin administration recapitulates hypertensive phenotypes by elevating blood pressure, upregulating ERS biomarkers, and concurrently activating IRE1α and PERK signalling axes [70]. In hypertensive environments, mechanical stress itself initiates a self‐perpetuating cycle of ERS induction and vascular remodelling, creating a positive feedback loop that exacerbates arteriopathy [71]. Collectively, these findings delineate ERS as a central regulator of VSMC fate, capable of driving both apoptotic loss and hyperproliferative remodelling in the hypertensive vasculature.

### 3.5 ERS and Cardiac Remodelling

While cardiac remodelling constitutes a cardinal manifestation of hypertensive target organ damage, it concurrently potentiates bidirectional disease progression through maladaptive feedback mechanisms [72]. Substantial evidence establishes ERS as a critical mediator of hypertension‐associated cardiac structural and functional alterations [34]. ERS contributes to cardiomyocyte apoptosis through specific signalling pathways, as demonstrated by the ATF6‐CHOP axis, which mechanistically promotes cell death in hypertensive models [34]. This apoptotic pathway is further activated in diverse hypertensive contexts, including salt‐sensitive paradigms where chronic high‐sodium intake induces concentric cardiac hypertrophy concomitant with marked upregulation of myocardial ERS biomarkers (GRP78, CHOP) [73], and in volume overload cardiomyopathy where ERS‐CHOP axis activation drives apoptotic cardiomyocyte loss [74].

Beyond apoptosis, ERS signalling integrates broader remodelling processes, with coordinated IRE1α/PERK phosphorylation contributing to both cardiomyocyte hypertrophic signalling and cardiac fibroblast activation [75]. The functional significance of ERS in cardiac pathology is further substantiated by intervention studies demonstrating that ERS attenuation correlates with improved cardiac outcomes: genetic or pharmacological approaches targeting ERS pathways consistently ameliorate maladaptive cardiac remodelling, attenuate apoptosis, and improve ventricular performance across various hypertensive models [34, 74, 75, 76]. Collectively, these findings establish ERS as a central pathological mechanism coordinating multiple aspects of hypertensive cardiac remodelling, from cardiomyocyte apoptosis to fibroblast activation and functional impairment.

**FIGURE 2:**
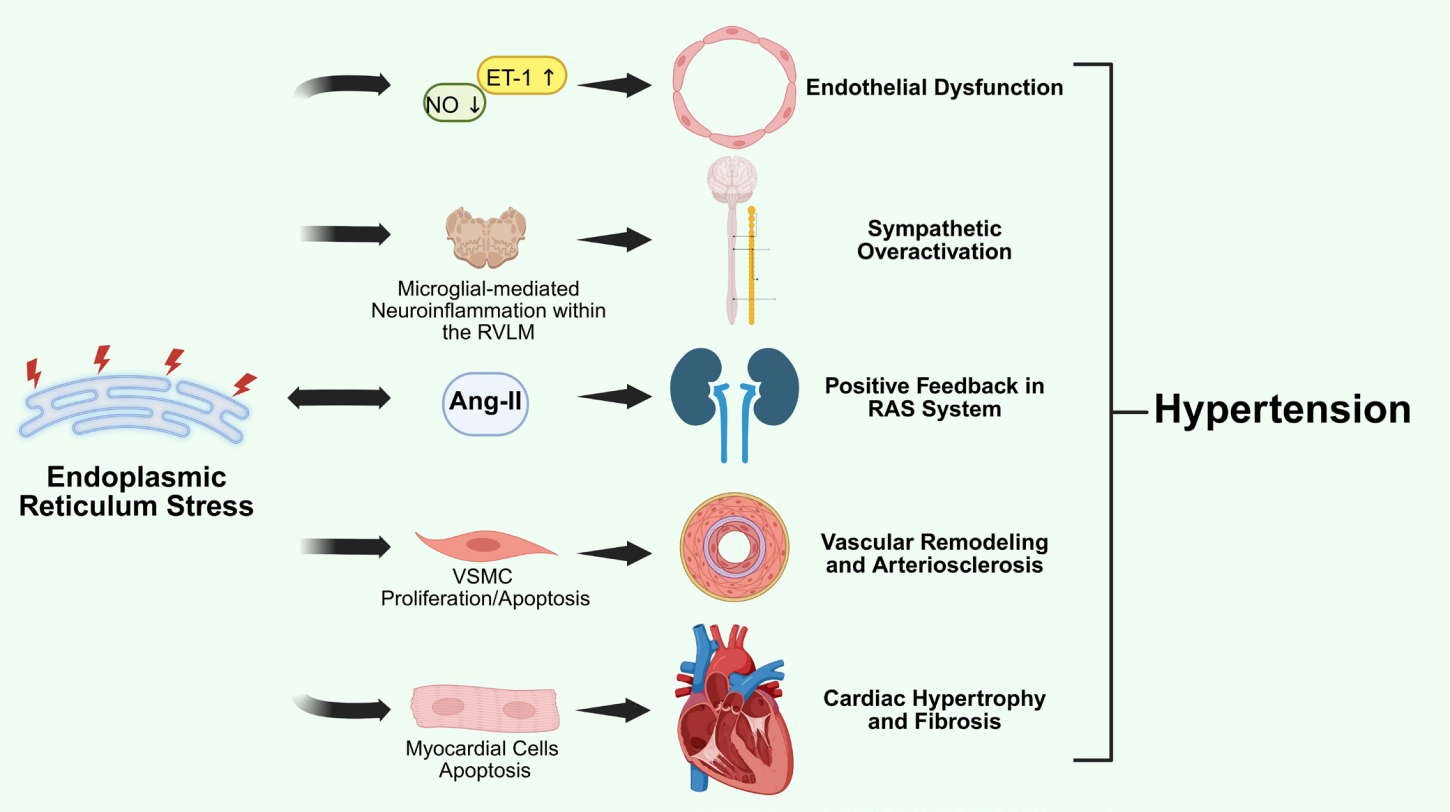
Mechanisms related to ERS induced hypertension. Endoplasmic reticulum stress (ERS) drives the development and progression of hypertension through multiple interconnected mechanisms. These include impairment of vascular endothelial function, aberrant proliferation of vascular smooth muscle cells leading to vascular remodelling, sympathetic nervous system overactivation, and a positive feedback loop within the renin‐angiotensin system. Furthermore, ERS contributes directly to cardiac injury, promoting hypertrophic and fibrotic remodelling. The convergence of these pathways thereby sustains the development and progression of hypertension. Ang‐II, Angiotensin II; ET‐1, Endothelin‐1; NO, Nitric Oxide; RVLM, Rostral Ventrolateral Medulla; VSMC, Vascular Smooth Muscle Cell.

Overall, the role of ERS in the pathogenesis and progression of hypertension‐related diseases primarily involves the aforementioned processes. Vascular endothelial dysfunction, abnormal proliferation/apoptosis of VSMCs, and the interplay between the SNS and the cardiovascular system collectively form the core pathological basis of these mechanisms (Figure 2).

## 4 Therapeutic Strategies Targeting ERS for Hypertension

### 4.1 Chemical Chaperones

Chemical chaperones constitute a class of low‐molecular‐weight compounds that orchestrate native protein folding trajectories and maintain structural fidelity of polypeptides [77], classified as one of three principal chaperonic systems alongside molecular chaperones and pharmacochaperones [78]. Accumulating preclinical evidence demonstrates that chemical chaperones dynamically regulate ERS activation dynamics and demonstrate translational potential across multiple pathological states, with emerging applications in hypertensive pathophysiology modulation.

#### 4.1.1 4‐Phenylbutyric Acid (4‐PBA)

4‐Phenylbutyric acid (4‐PBA), a pharmacological chaperone [79] and pharmacologically validated ERS inhibitor [80, 81], attenuates ERS through dual mechanisms of protein conformational stabilisation and suppression of misfolded polypeptide aggregation [82]. Critically, 4‐PBA (and its prodrug sodium phenylbutyrate, NaPB) has an established safety profile from decades of use in urea cycle disorders and other inborn errors of metabolism, demonstrating its tolerability for chronic administration in human patients [83]. This compound concomitantly mediates pleiotropic cytoprotective effects encompassing antioxidative defence mechanisms, anti‐inflammatory modulation, and inhibition of pro‐apoptotic cascades [84]. Supporting its potential metabolic benefits in hypertensive patients—who frequently exhibit insulin resistance—a randomised controlled trial in type 2 diabetes demonstrated that NaPB treatment significantly improved peripheral insulin sensitivity by 27% and reduced plasma glucose levels [85].

Extensive preclinical evidence further supports 4‐PBA's efficacy in hypertensive models. In murine models of diet‐induced metabolic dysfunction, 4‐PBA administration significantly attenuates ERS‐associated biomarker expression (GRP78, CHOP), mitigates oxidised low‐density lipoprotein (ox‐LDL)‐mediated ROS generation in VSMCs, and suppresses VSMC apoptotic clearance [86]. Additionally, 4‐PBA demonstrates therapeutic efficacy in ameliorating hypoxia‐induced pathophysiological alterations in pulmonary arterial smooth muscle cells [87]. In hypertensive contexts, 4‐PBA intervention reduces hypertensive indices in rodent models through coordinated mechanisms: downregulation of aortic ERS markers (including CHOP), restoration of nitric oxide (NO) bioavailability, and transcriptional upregulation of endothelial nitric oxide synthase (eNOS) [88]. In spontaneously hypertensive rat (SHR) paradigms, chronic 4‐PBA treatment elicits significant reductions in systolic and diastolic blood pressure parameters, though incomplete normalisation to normotensive baselines [89]. Furthermore, 4‐PBA administration diminishes ERS biomarker expression in SHR mesenteric resistance vessels, enhances endothelium‐dependent vasorelaxation, and reduces vascular tone through smooth muscle hyperpolarization [90]; it also rebalances renal RAS components and rescues pressure‐natriuresis, contributing to systemic blood pressure reduction [91].

The anti‐inflammatory properties of 4‐PBA observed in preclinical models are also recapitulated in human studies. In amyotrophic lateral sclerosis patients, combination therapy containing sodium phenylbutyrate significantly reduced plasma levels of the pro‐inflammatory biomarkers YKL‐40 and C‐reactive protein (CRP) [92]. By orchestrating concurrent suppression of ER proteotoxicity, oxidative stress burden, apoptotic signalling, and inflammation—as evidenced across both preclinical and emerging human studies—4‐PBA exhibits robust vasculoprotective properties against hypertensive vascular pathophysiology. These collective data therefore support its investigation as a novel therapeutic agent for hypertension management, and future clinical trials in hypertensive populations would help to definitively establish its efficacy.

#### 4.1.2 Tauroursodeoxycholic Acid (TUDCA)

Tauroursodeoxycholic acid (TUDCA), an endogenous bile acid derivative and pharmacologically active constituent of ursodeoxycholic formulations, mediates cytoprotective effects through attenuation of pathological ERS activation [93] and suppression of apoptotic signalling cascades [94]. Critically, TUDCA has demonstrated a favourable safety and tolerability profile in human clinical trials across multiple chronic disease states, supporting its potential for repurposing in cardiovascular contexts [95]. Preclinical investigations substantiate TUDCA's capacity to ameliorate systemic metabolic dysregulation via dual modulation of ERS and inflammatory pathway inhibition [96].

In the context of hypertension, TUDCA demonstrates multi‐organ protective efficacy through several distinct yet complementary mechanisms. In salt‐sensitive hypertensive models, TUDCA administration elicits significant reductions in systolic blood pressure through targeted ERS suppression [97], while concurrently attenuating hepatic inflammatory infiltrates and fibrotic remodelling [98]. Mechanistically, TUDCA improves vascular endothelial dysfunction through tripartite actions: restoration of NO bioavailability, upregulation of sarco/endoplasmic reticulum Ca^2+^‐ATPase (SERCA) expression, and normalisation of ER calcium cycling dynamics [99]. Cardiac‐specific protection is evidenced by TUDCA's ability to restore cardiac proteostatic equilibrium by modulating GRP78, SERCA, NF‐κB p65, and Bcl‐2 expression profiles, thereby rectifying ERS‐associated calcium dysregulation, inhibiting mitochondrial apoptosis, and attenuating inflammatory myocardial injury [97].

The translational relevance of these mechanisms is supported by emerging human data. In patients with ulcerative colitis, a condition characterised by inflammation and tissue injury, TUDCA supplementation significantly reduced endoscopic and histological markers of ERS and inflammation, concurrently promoting tissue healing and improving clinical disease activity [100]. This demonstration of TUDCA's capacity to mitigate ER stress and inflammation in human pathophysiology provides a compelling rationale for its investigation in hypertensive organ damage.

Furthermore, TUDCA intervention demonstrates efficacy across various cardiac stress models, mitigating pressure overload‐induced cardiomyocyte apoptosis and pathological hypertrophy [76], as well as restoring proteostatic balance and improving ventricular systolic performance in volume overload cardiomyopathy [74]. Cumulatively, TUDCA establishes its therapeutic potential as a pleiotropic intervention for hypertension through integrated mechanisms—proteostatic regulation of ERS, calcium homeostasis restoration, anti‐apoptotic signalling, and anti‐inflammatory modulation—targeting multiple end‐organ pathologies simultaneously. The concordance between robust preclinical evidence in hypertension models and established safety and biological activity in human trials positions TUDCA as a promising candidate for future clinical evaluation in cardiovascular diseases.

### 4.2 Antioxidants

Emerging evidence has characterised reciprocal pathophysiological interactions between oxidative stress and ERS, proposing that antioxidant‐mediated attenuation of redox imbalance may confer indirect mitigation of ERS‐associated cellular dysregulation [101].

#### 4.2.1 N‐Acetylcysteine (NAC)

N‐Acetylcysteine (NAC), a thiol‐based antioxidant with established clinical utility, is employed in the therapeutic management of respiratory disorders characterised by pathological mucus hypersecretion, including chronic obstructive pulmonary disease (COPD) and bronchiectasis [102]. Its extensive clinical use has established a well‐characterised safety profile for both acute and chronic administration in humans. Mechanistic studies reveal that NAC attenuates ROS‐mediated ERS activation, consequently suppressing alveolar epithelial cell apoptosis in murine COPD paradigms [102]. In hyperglycemic cardiomyocyte injury models, combination therapy with insulin and NAC significantly downregulates ERS biomarkers (GRP78, IRE1α, CHOP) and apoptotic regulators (Caspase‐3, Bax), indicative of its cardioprotective efficacy through coordinated ERS suppression [103].

The translational potential of NAC's organoprotective mechanisms is supported by human clinical evidence. In patients undergoing double‐valve cardiac replacement surgery—a setting of profound oxidative stress and inflammation—prophylactic NAC administration significantly reduced postoperative liver dysfunction and shortened the duration of intensive care unit stay [104]. This finding demonstrates NAC's capacity to mitigate end‐organ injury in a clinically relevant cardiovascular context.

Advanced delivery systems utilising nanoparticle‐encapsulated NAC enhance therapeutic potency against hypoxia/reoxygenation‐induced cardiomyocyte ultrastructural damage and programmed cell death [105]. In SHR models, NAC intervention demonstrates multifaceted benefits: normalisation of oxidative stress markers, preservation of cardiac functional architecture [106], and partial attenuation of hypertensive phenotypes [107]. Notably, maternal NAC supplementation in SHRs prevents systolic hypertension transmission to male progeny, potentially mediated through modulation of hydrogen sulphide (H_2_S) biosynthesis pathways and gut microbiota dysbiosis [107].

Furthermore, the efficacy of NAC in modulating pathophysiological processes in human disease is reinforced by a clinical trial in hereditary cystatin C amyloid angiopathy, where high‐dose NAC treatment was well‐tolerated and significantly reduced disease‐specific biomarker levels [108]. Through dual inhibition of oxidative stress and ERS signalling cascades, NAC exhibits pronounced cytoprotective properties across diverse pathophysiological contexts, underscoring its translational potential as a multimodal therapeutic agent for hypertension and associated end‐organ pathologies.

#### 4.2.2 Melatonin

Melatonin (MT), an indoleamine neurohormone secreted by the pineal gland, demonstrates significant therapeutic potential through its potent free radical‐scavenging antioxidant properties and regulatory effects on ERS pathophysiology [109]. Compelling human evidence supports its role in blood pressure regulation. A randomised controlled trial in normotensive adults on a high‐sodium diet demonstrated that melatonin supplementation specifically reduced nighttime peripheral and central systolic blood pressure, indicating its potential to modulate hemodynamic parameters in humans [110]. Furthermore, in a unique clinical model of melatonin deficiency—pinealectomized patients—melatonin replacement therapy significantly improved cardiovascular parameters, including reduced diastolic blood pressure, suggesting a direct physiological role in human cardiovascular homeostasis [111].

The mechanisms underlying these clinical observations are elucidated by preclinical studies. In nickel‐induced cardiotoxicity models, MT administration restores cardiac expression profiles of ERS‐associated biomarkers (ATF‐4, IRE1α, CHOP) and apoptotic regulators (Caspase‐3, Bcl‐2, Bax), mechanistically confirming its cardioprotective efficacy via suppression of ERS‐mediated cardiomyocyte apoptosis [112]. Experimental evidence substantiates MT's capacity to ameliorate endothelial dysfunction through ERS attenuation, mediated through suppression of ERS‐induced apoptotic signalling cascades [109].

The translational relevance of melatonin's antioxidant and anti‐inflammatory properties is further strengthened by human interventional studies. In patients undergoing coronary artery bypass grafting (CABG), postoperative melatonin administration significantly improved left ventricular ejection fraction and reduced systemic oxidative stress and inflammatory biomarkers, demonstrating direct cardioprotective effects in a clinical setting of intense oxidative stress [113].

The MT derivative ITH3001 exhibits antihypertensive and cardioprotective effects in murine angiotensin II infusion models, ameliorating aortic endothelial dysfunction via coordinated suppression of oxidative stress and inflammatory activation. This is evidenced by significant attenuation of pro‐inflammatory mediators including interleukin‐1β (IL‐1β), interleukin‐6 (IL‐6), nuclear factor‐κB p65 (NF‐κB p65), and NLRP3 inflammasome activity [114, 115]. Collectively, MT orchestrates modulation of ERS‐related signalling networks to inhibit pathological ERS activation. The convergence of evidence from well‐designed human trials—demonstrating direct blood pressure modulation and organ protection—with robust preclinical mechanistic data, solidifies melatonin's position as a critically relevant modulator with multiscale cellular protective effects, warranting its further investigation as an adjunctive therapy for hypertension.

### 4.3 Novel Mechanisms of Traditional Antihypertensive Drugs

#### 4.3.1 Angiotensin‐Converting Enzyme Inhibitors (ACEIs)

Angiotensin‐converting enzyme inhibitors (ACEIs) mediate antihypertensive and organoprotective effects through inhibition of ACE enzymatic activity and suppression of angiotensin II (Ang II) biosynthesis. Emerging evidence substantiates that ACEIs exert additional organ‐protective properties via attenuation of ERS‐associated signalling pathways. Experimental investigations demonstrate that captopril potentiates morphine analgesia while preventing analgesic tolerance through suppression of ERS activation in rodent dorsal root ganglia [116]. Combinatorial therapy utilising benazepril and moxibustion significantly inhibits cardiac ERS biomarkers, including phosphorylated PERK (p‐PERK) and phosphorylated eukaryotic initiation factor 2α (p‐eIF2α), thereby ameliorating chronic heart failure progression in murine models. This therapeutic regimen enhances left ventricular ejection fraction (LVEF) and fractional shortening indices, reduces circulating brain natriuretic peptide (BNP) concentrations, and improves global cardiac functional parameters [117]. Furthermore, methionine‐enriched dietary regimens induce ACE‐dependent aortic ERS, exacerbating hypertensive pathogenesis and potentiating Ang II‐mediated aortic contractile hyperresponsiveness in rodent models. Enalapril administration attenuates aortic ERS through dual inhibition of PERK and eIF2α phosphorylation, resulting in significant blood pressure reduction [118]. In hypertensive rodent systems, enalapril suppresses VSMC expression of GRP78 and CHOP, alleviates VSMC ERS burden, reduces arterial medial hypertrophy, and improves vascular architectural remodelling—mechanisms collectively contributing to its antihypertensive efficacy [119].

The translational significance of these findings is underscored by considering the established clinical profile of ACEIs. Their proven efficacy in humans—such as regressing left ventricular hypertrophy, improving endothelial function, and reducing clinical heart failure events—directly aligns with the correction of ERS‐driven pathological processes, including maladaptive hypertrophy, apoptosis, and inflammation, as demonstrated in preclinical models. These findings elucidate that ACEIs not only reduce blood pressure via RAAS inhibition but also ameliorate cardiovascular pathophysiology through ERS modulation, thereby positioning ERS suppression as a plausible and complementary mechanism contributing to their well‐documented clinical benefits.

#### 4.3.2 Angiotensin II Receptor Blockers (ARBs)

Angiotensin receptor blockers (ARBs) exert antihypertensive effects through competitive inhibition of angiotensin II (Ang II) binding to the angiotensin type 1 receptor (AT1R), thereby antagonising Ang II‐mediated pathophysiological signalling. Accumulating evidence indicates that ARBs additionally mediate organoprotective effects via suppression of ERS activation. Preclinical studies demonstrate that candesartan attenuates cardiomyocyte ERS, reducing caspase‐dependent apoptosis and inhibiting the progression of dilated cardiomyopathy in experimental models [120]. Similarly, irbesartan ameliorates diabetes‐associated cardiac structural and functional alterations by normalising ERS hyperactivation in diabetic rodent cardiomyocytes [121]. Chronic intermittent hypoxia induces endothelial ERS and apoptotic clearance, whereas losartan intervention mitigates hypoxia‐induced ERS, preserves endothelial viability, and restores vasoregulatory function [122]. Homocysteine induces ERS activation in endothelial cells, leading to damage. Valsartan can alleviate homocysteine‐induced ERS in endothelial cells [123]. Telmisartan demonstrates metabolic benefits through AMP‐activated protein kinase (AMPK)‐dependent ERS inhibition, attenuating obesity‐induced insulin resistance [124].

The clinical relevance of these mechanisms is supported by the established role of ARBs in reducing end‐organ damage in human hypertension, effects that align with the attenuation of ERS‐driven pathology observed preclinically. These findings collectively elucidate that ARBs not only reduce blood pressure via AT1R blockade but also enhance cardiovascular and metabolic homeostasis through ERS modulation, providing dual therapeutic modalities for hypertension management and its multisystemic complications.

## 5 Conclusions and Perspectives

Contemporary advancements in scientific methodologies and heightened understanding of cardiovascular pathophysiology have propelled substantial progress in elucidating the molecular mechanisms underlying hypertension—a pervasive global health challenge. It is now recognised that ERS plays a multifaceted and complex role in hypertension pathogenesis. Through evolutionarily conserved signalling axes including the IRE1α‐XBP1s cascade, ATF6‐CHOP pathway, and PERK‐eIF2α phosphorylation network, ERS orchestrates multifaceted cellular responses encompassing apoptotic execution, oxidative stress amplification, inflammatory cytokine production, and calcium dyshomeostasis. These molecular perturbations collectively drive hallmark hypertensive pathologies: vascular endothelial dysfunction, aberrant VSMC proliferation, RAS hyperactivation, and sympathetic hyperactivation, ultimately culminating in vascular remodelling, myocardial hypertrophy, and multi‐organ damage. Sustained, unresolved ERS induces maladaptive hyperactivation of these pathways, exacerbating cellular proteotoxic stress and accelerating hypertensive pathogenesis. However, the mechanistic interplay between ERS and emerging etiological factors (e.g., anti‐neoplastic therapy‐induced hypertension) remains insufficiently characterised. Furthermore, the development of clinically applicable biomarkers for quantifying ERS intensity and its temporal correlation with disease staging constitutes a critical unmet need.

Pharmacological modulation of ERS emerges as a promising therapeutic frontier in hypertension management (Table 1). The strategy of drug repurposing, exemplified by chemical chaperones and antioxidants, is particularly promising given their established safety profiles and the emerging human data supporting their biological efficacy. Chemical chaperones (e.g., 4‐PBA, TUDCA) and redox‐modulating compounds (e.g., NAC, melatonin) demonstrate robust antihypertensive and organoprotective efficacy in preclinical models through ERS attenuation, with their translational potential now being progressively supported by initial clinical findings. Notably, conventional antihypertensive agents—including ACEIs and ARBs—exhibit ancillary ERS‐suppressive properties, thereby expanding their therapeutic potential. Nevertheless, current evidence remains predominantly derived from experimental models, necessitating rigorous clinical validation through multicenter trials and mechanistic studies in human cohorts.

**TABLE 1:** Pharmacological targeting of ERS in hypertension.

Category	Agents	Key mechanisms	Associated pathological contexts/diseases	References
Chemical chaperones	4‐PBA/NaPB	Attenuates ERS via proteostasis; reduces ERS‐mediated apoptosis	Endothelial dysfunction, VSMC dysregulation, hypertension	[82, 86, 88, 89, 90]
	TUDCA	Direct ERS inhibition; ameliorates ERS‐driven calcium dysfunction	Salt‐sensitive hypertension, cardiac remodelling, vascular injury	[74, 93, 97, 99]
Antioxidants	N‐Acetylcysteine (NAC)	Reduces oxidative stress & ERS; suppresses ERS‐apoptosis axis	Hypertension transmission, diabetic cardiotoxicity	[102, 103, 106, 107]
	Melatonin	Antioxidant‐mediated ERS suppression; blocks ERS‐apoptosis	Endothelial dysfunction, nighttime hypertension	[109, 110, 111, 112, 114]
Conventional antihypertensives (novel mechanisms)	ACEIs (e.g., Enalapril)	Suppresses ERS (PERK pathway); improves ERS‐associated remodelling	Hypertensive vascular remodelling, heart failure	[117, 118, 119]
	ARBs (e.g., Valsartan)	Alleviates ERS; attenuates ERS‐induced apoptosis	Diabetic cardiomyopathy, endothelial damage	[120, 121, 122, 123]

*Note:* Summary of agents that ameliorate hypertension and related organ damage by modulating endoplasmic reticulum stress (ERS).

Future investigative priorities should concentrate on: (1) Deciphering cross‐regulation between ERS and hypertension‐associated signalling networks (e.g., RAS, sympathetic‐adrenal‐medullary axis) to delineate its spatiotemporal contributions to disease pathogenesis; (2) Developing isoform‐selective ERS modulators targeting IRE1α ribonuclease activity, PERK kinase function, or ATF6 proteolytic processing, potentially integrated with CRISPR/Cas9‐mediated genomic editing for precision interventions; (3) Advancing translational research to evaluate ERS‐targeted therapies in hypertensive populations, with concurrent assessment of safety profiles and exploration of personalised treatment algorithms. Furthermore, the emerging role of ERS in oncology therapy‐associated hypertension warrants systematic investigation into its mechanistic underpinnings. Through interdisciplinary collaboration integrating molecular biology, pharmacotherapy, and clinical epidemiology, ERS modulation may emerge as a transformative therapeutic strategy—offering innovative approaches to optimise clinical outcomes, enhance life quality, and reduce hypertension‐related morbidity.

## Author Contributions

**Xin Ma:** conceptualization, writing – original draft (lead), writing – review and editing (lead), visualisation. **Fei Si:** writing – review and editing (supporting). **Jie Ma:** supervision. **Chuyan Feng:** supervision. **Yingdong Wang:** supervision. **Luosha Wang:** supervision. **Jing Yu:** funding acquisition (lead), project administration (lead), writing – review and editing (supporting). All authors contributed to the article and approved the submitted version.

## Funding

This study was supported by the National Natural Science Foundation of China (NSFC 81960086, 82160089, 82460086), and the Cuiying Scientific and Technological Innovation Program of Lanzhou University Second Hospital (CY2021‐MS‐A13). This study was also supported by the Special Fund Project for Doctoral Training of the Lanzhou University Second Hospital (YJS‐BD‐24) and the International Science and Technology Cooperation Base (PR0124002).

## Disclosure

Human and animal rights and informed consent: This article does not contain any studies with human or animal subjects performed by any of the authors. This author takes responsibility for all aspects of the reliability and freedom from bias of the data presented and their discussed interpretation.

## Ethics Statement

The authors have nothing to report.

## Conflicts of Interest

The authors declare no conflicts of interest. Figures were created with biorender.com.

## Data Availability

The authors have nothing to report.
